# Management of extensive portal vein thrombosis via thrombolysis and thrombectomy without underlying liver disease: A case report

**DOI:** 10.1002/ccr3.8920

**Published:** 2024-07-01

**Authors:** Mohammadshah Isam Gul, Waseem Umer, Ahmed Daniyal Nawaz, Mohammad J. H. Elhissi, Muhammad Zahid

**Affiliations:** ^1^ Internal Medicine Department Hamad Medical Corporation Doha Qatar; ^2^ Qatar University Doha Qatar; ^3^ Radiology Department Hamad Medical Corporation Doha Qatar; ^4^ Weill Cornell Medicine Doha Qatar

**Keywords:** anticoagulation, case report, liver cirrhosis, portal vein thrombosis, thrombectomy, thrombolysis

## Abstract

Portal vein thrombosis (PVT) is a rare condition, particularly in non‐cirrhotic patients. Anticoagulation remains the mainstay of the treatment. Extensive PVT can lead to variceal bleeding, ascites, bowel ischemia, and hypersplenism. The role of thrombolysis and thrombectomy in these patients remains unclear. However, there is evidence that local thrombolysis and thrombectomy should be considered in those who remain symptomatic on anticoagulation and are at risk of complications with acute PVT.

## 1 INTRODUCTION

The splenic vein (SV) and superior mesenteric vein (SMV) combine to form a portal vein draining the spleen and small intestine, respectively. The portal vein constitutes roughly 75% of the blood flow to the liver.^1^ Portal vein thrombosis (PVT) can be acute or chronic, complete or partial, and may involve a thrombus extending into the SV and SMV.

The PVT is a rare condition with a prevalence of 1/100,000.^2^, ^3^, ^4^ PVT commonly occurs in cirrhotic and liver transplant patients.^5^ Non‐cirrhotic‐related PVT occurs in the setting of local conditions (like cholangitis, pancreatitis, diverticulitis, and abdominal trauma), systemic diseases (like myeloproliferative disorders (MPS), paroxysmal nocturnal hemoglobinuria (PNH), antiphospholipid syndrome (APS)), or malignancy (like hepatic, gastric, and pancreatic cancer).^6^, ^7^

The complications of PVT are variceal bleeding, ascites, bowel ischemia, splenomegaly, hypersplenism, and hepatic encephalopathy in patients with liver cirrhosis.^8^ Morbidity and mortality associated with PVT depend on the underlying cause. GI bleeding with PVT without underlying liver disease at 2 years is 0.25%, while it is >50% in patients with liver cirrhosis. With anticoagulant therapy, the prognosis is good, and 10‐year survival is >70%.^8^, ^9^, ^10^ Anticoagulation is the mainstay of treatment for PVT, but for patients with extensive PVT who remain symptomatic, thrombolysis and thrombectomy are also considered in acute settings to prevent complications. The indications for thrombolysis and thrombectomy for PVT are unclear in the literature. We present a case of extensive PVT with SMV and SV extension, remaining symptomatic despite oral anticoagulation; hence, thrombolysis and thrombectomy were done.

## 2 CASE HISTORY/EXAMINATION

A 35‐year‐old Filipino lady presented to the emergency department of Hamad General Hospital, Qatar, with a chief complaint of abdominal pain for 3 days before admission. The pain was in the central area (epigastric, periumbilical, suprapubic), 5/10 in severity, continuous, and sometimes radiates to the back. There were no relieving or aggravating factors. She denied nausea, vomiting, or a change in bowel habits. She also negated melena or bleeding per rectum, vaginal bleeding or discharge, dysuria, or hematuria. There was no previous history of similar attacks. She complained of intermittent shortness of breath in the last 3 days but denied chest pain, palpitations, orthopnea, or paroxysmal nocturnal dyspnea. She denied fever, joint pain or swelling, and lower limb pain or swelling. She had no significant past medical or surgical history. She smoked a pipe 1–2 times weekly and drank alcohol occasionally, mainly on weekends. She did not have any allergies. She had not taken any medications regularly except for OCP (Diane – estrogen/progesterone combination) for the past 4 years. The family history was positive for the mother's liver cancer, but she denied any family history of thrombophilia.

On examination, she had a pulse of 86/min, a blood pressure of 125/78 mmHg, a respiratory rate of 20/min, and maintained an oxygen saturation of 98% on room air. She had mild tenderness in the right half of her abdomen without guarding or rigidity. Chest, cardiovascular, and neurological examinations were essentially normal. There was no swelling in the legs.

## 3 METHODS

Based on the history and examination, our differentials were pancreatitis, hepatitis and bleed in the GI tract. Routine investigations showed a normal complete picture and serum lipase, liver, renal, and thyroid functions except raised CRP 92.1 mg/L, albumin 33 gm/L, and K 3.4 mmol/L (Table 1).

**TABLE 1:** Complete blood count and comprehensive metabolic panel on admission.

	Result	Reference
WBC	11.8 × 10^3^/μL	4–10 × 10^3^/μL
Absolute Neutrophil count (ANC)	8.9 × 10^3^/μl	2.0–7.0 × 10^3^/μL
Lymphocyte	1.7 × 10^3^/μL	1–3 × 10^3^/μL
Monocyte	1.0 × 10^3^/μL	0.2–1.0 × 10^3^/μL
Eosinophil	0.10 × 10^3^/μL	0.02–0.5 × 10^3^/μL
Basophil	0.08 × 10^3^/μL	0.02–0.10 × 10^3^/μL
Hgb	13.5 gm/dL	12–15 gm/dL
Hct	40.9%	36.0–46.0%
MCV	84 fL	83–101 fL
Platelets	412 × 10^3^/μL	150–410 × 10^3^/μL
Urea	2.5 mmol/L	2.5–7.8 mmol/L
Creatinine	70 μmol/L	44–80 μmol/L
Sodium	136 mmol/L	133–146 mmol/L
Potassium	3.4 mmol/L	3.5–5.3 mmol/L
chloride	100 mmol/L	95–108 mmol/L
Bicarbonate	23 mmol/L	22–29 mmol/L
Calcium	2.25 mmol/L	2.20–2.60 mmol/L
Bilirubin T	7 μmol/L	0–7 μmol/L
Albumin	33 gm/L	35–50 gm/L
Alkaline phosphate	79 U/L	35–104 U/L
ALT	28 U/L	0–33 U/L
AST	26 U/L	0–32 U/L
Lipase	31 U/L	16–60 U/L
CRP	92.1 mg/L	0.0–5.0 mg/L
Beta hCG	Negative	

Ultrasonography (USG) of the abdomen showed a normal liver, no sonographic evidence of liver disease, and a normal‐sized spleen. Both kidneys, pancreas, and gallbladder looked normal. There were no ascites. The portal vein was 10.2 mm in diameter; no flow was noted, raising the possibility of portal vein thrombosis.

A CT scan of the abdomen with contrast showed non‐opacification, lumen expansion, and stranding of the adjacent surrounding fat involving the entire SMV, part of SV, and entire main PV, extending to involve the main right and left PV, and their tributes, which were highly suggestive of extensive acute thrombosis. Major visceral organs in the abdomen and pelvis, major vessels, and bony structures were unremarkable, and no free fluid was noted.

Further workup was arranged to look for underlying causes of acute PVT. The pro‐thrombotic state tests returned negative results (Table 2). The screening tests for PNH, autoimmune disease, and APS also returned negative (Table 3). The use of oral contraceptive pills was considered the most likely reason for her extensive acute PVT. She was started on a therapeutic dose (1 mg/Kg q12hr) of low molecular weight heparin (LMWH).

**TABLE 2:** Coagulation profile and thrombophilia workup.

	Result	Reference
APCR	0.96	0.91–1.19
PTT	12.2 sec	9.4–12.5 s
APTT	29.5 sec	25.1–36.5 s
INR	1.1	
D‐dimer	126 mg/L	0–49 mg/L
Fibrinogen	4.49 gm/L	2–4.1 gm/L
Protein C	114%	70–140%
Protein S	79.4%	56–126%
Homocysteine plasma LC‐MSMS	9.1	0–13
Factor II	Negative	
Factor V Leiden	Negative	

**TABLE 3:** Autoimmune workup.

	Results	Titers	Reference
Anti‐nuclear antibodies	Negative		
Anti dsDNA Ab	Negative	1.40 IU/mL	<10
Anti‐neutrophil cytoplasmic antibody	Negative		
Anti cardiolipin Ab IgG	Negative	1.40 GPL	<10
Anticardiolipin Ab IgM	Negative	0.90 MPL	<10
Anti B2 glycoprotein IgG	Negative	1.10 U/mL	<7
Anti B2 glycoprotein IgM	Negative	<2.90 U/mL	<7
Lupus ratio	Negative	1.29	1.01–1.41

## 4 TREATMENT

**FIGURE 1:**
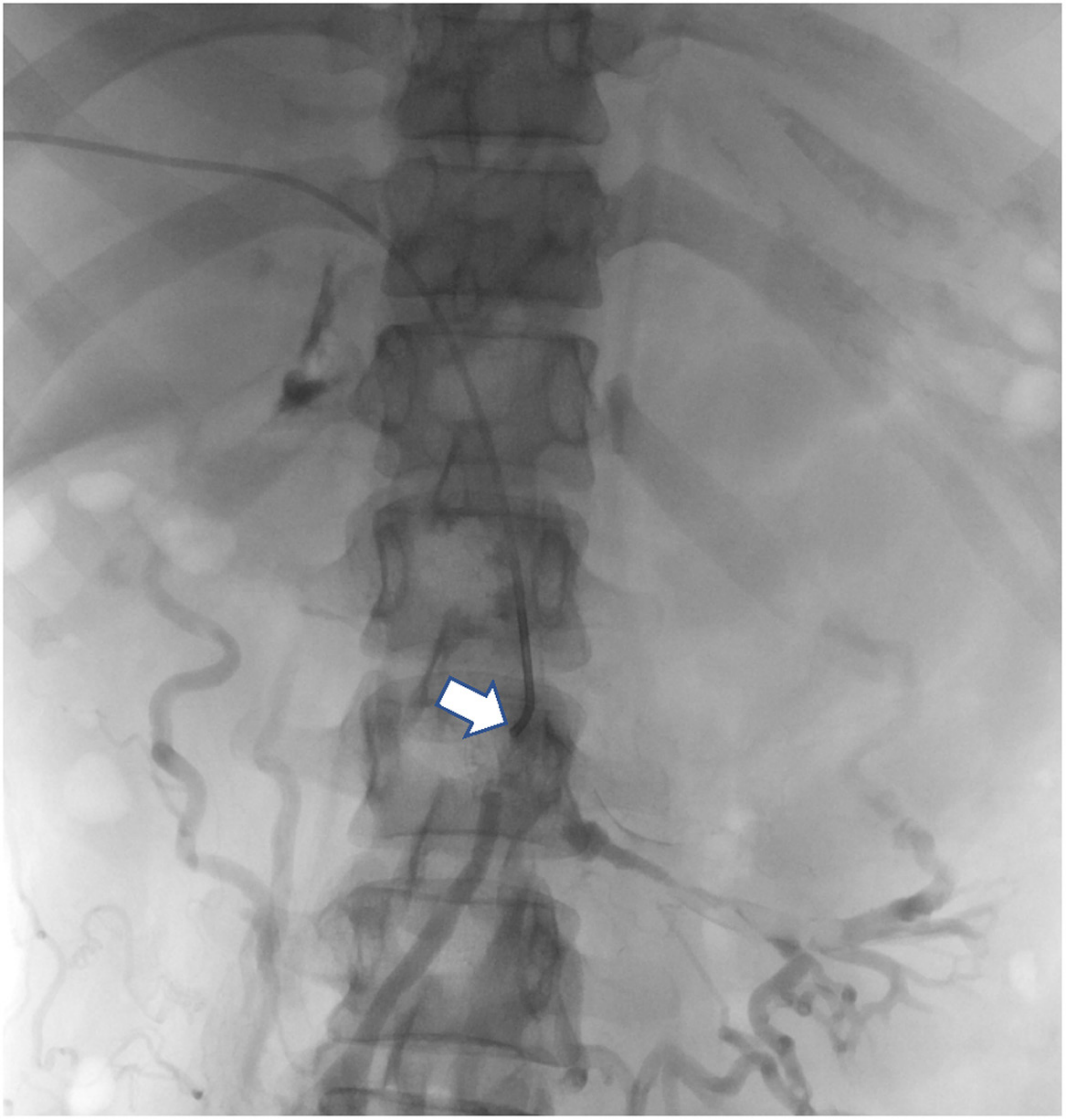
Pre‐thrombectomy portal venogram; the tip of the catheter (arrow) is in the SMV with the retrograde flow of contrast with collaterals.

**FIGURE 2:**
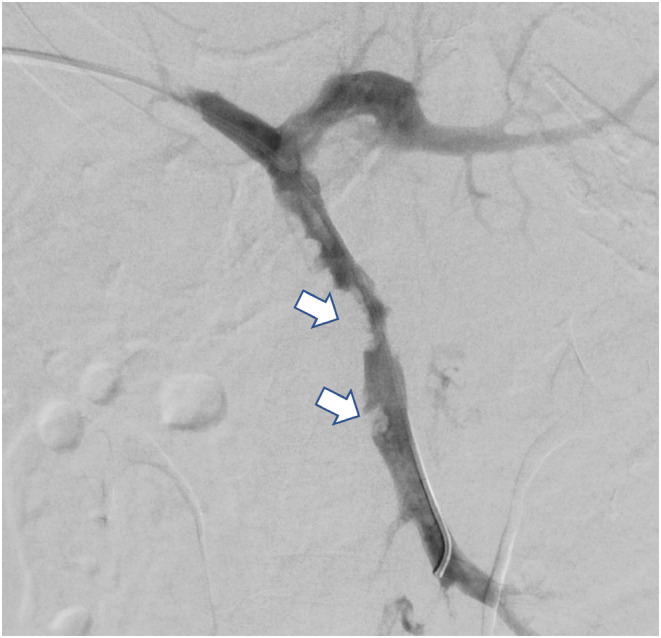
A post‐thrombectomy portal venogram; the tip of the catheter is in the SMV with the antegrade flow of contrast with the disappearance of collaterals. A few remnant thrombi (Arrows) persist in the SMV.

She remained symptomatic with continuous abdominal pain (8–9/10 severity) even with regular analgesics and nausea and vomiting despite being on therapeutic anticoagulation. Given extensive thrombosis, persistent symptoms, and the risk of complications like mesenteric ischemia, hypersplenism, and sepsis, an interventional radiologist's opinion was taken, and we planned for thrombolysis and mechanical thrombectomy. The pros and cons of the procedure were discussed with the patient, who agreed to proceed with thrombolysis/thrombectomy (Figures 1 and 2). Five days after her initial presentation and being on therapeutic anticoagulation, she was planned for thrombolysis/thrombectomy via interventional radiology.

## 5 PROCEDURE TECHNIQUE AND CONCLUSION

The thrombosed right deep portal vein branch was punctured under CT guidance, and the wire passed smoothly, confirming a fresh thrombus. Subsequent angiogram confirmed extensive thrombus of the SMV and PV with partial flow in the left PV branches. The thrombus was cleared using an Angioget machine followed by a tissue plasminogen (tPA) infusion.

After that, the venogram showed improvement, and around 80% improvement was deemed reasonable, which will improve further after resuming the therapeutic dose of anticoagulation. She remained stable after that, with no overt signs of bleeding. Post‐procedure ultrasound did not show bleeding or hematoma.

Her pain improved remarkably after the procedure. She was counseled on stopping OCP and choosing an alternative method of contraception.

## 6 FOLLOW‐UP

She was discharged on rivaroxaban for 3 months. She was followed up regularly for up to 18 months in the internal medicine clinic with no recurrence of symptoms.

## 7 DISCUSSION

Portal vein thrombosis is a rare disease, with an incidence of approximately 1% in the general population. PVT is more common in patients with cirrhosis, reported around 0.6%–16%, and the incidence increases in patients with advanced cirrhosis, being elevated to 5%–25% in liver transplantation cases and 40% in hepatocellular cancer patients. The incidence is rising as more abdominal imaging is done in the emergency department.^11^, ^12^, ^13^

Common conditions associated with PVT other than liver cirrhosis include PNH, MPS, inherited thrombophilia (Protein C&S deficiency), autoimmune diseases like APS, acquired thrombophilia (pregnancy and medications), and inflammatory conditions like inflammatory bowel disease.^14^

The clinical presentation of PVT depends upon the underlying condition and location of the thrombus, the degree of portal vein occlusion, and the extension into SMV and/or SV. The presentation of PVT can be acute or chronic. An acute presentation is usually symptomatic, including abdominal pain, nausea, and vomiting. Extensive thrombosis in the mesenteric venous system can cause bowel ischemia, which presents with pain, vomiting, rectal bleeding, and rarely sepsis. Chronic PVT can be asymptomatic, diagnosed either on abdominal imaging for unrelated problems or during investigation for portal hypertension or ascites.^14^

Our patient presented with severe epigastric pain radiating to the back, associated with nausea and vomiting. The initial workup ruled out common conditions like pancreatitis, biliary tract disease, pericarditis, and basal pneumonia. Ultrasound raised the suspicion of PVT, further confirmed with contrast‐enhanced computed tomography imaging. There was no evidence of chronic liver disease on examination or imaging. Further workup, including thrombophilia state, APS, PNH, and MPS screening, was carried out to look for an underlying cause, and the results were negative. As she was taking oral contraceptive pills, we concluded that she has extensive PVT secondary to the use of OCPs. Women using OCPs have a four‐fold increased risk of venous thromboembolism compared to those not using hormonal pills, as the use of OCPs alters the procoagulant factors and endogenous anticoagulant proteins in the body.^15^, ^16^

The American Association for the Study of Liver Diseases (AASLD) 2009 guideline recommends anticoagulation for patients with acute PVT without underlying liver disease.^17^ American College of Gastroenterology (ACG) clinical guidelines recommend anticoagulation for all PVT patients without liver cirrhosis as a first line of therapy in the absence of any contraindication, such as active bleeding. They recommend anticoagulation for at least 6 months for reversible causes of PVT, such as trauma or acute intra‐abdominal process without thrombophilia. However, an indefinite period of anticoagulation is recommended for PVT patients with thrombophilia.^18^

Portal vein thrombosis is treated with LMWH in the acute phase, followed by a vitamin K antagonist. Currently, the data on direct oral anticoagulants (DOACs) is limited,^18^ although more favorable results have recently been reported in the literature with DOACs in PVT. A systematic review compared different anticoagulants in patients with PVT and found that the resolution of PVT in the rivaroxaban group was 85% compared to 45% in the warfarin group. The short‐term survival of 20.4 ± 2.2 months in the rivaroxaban group was better than 10.6 ± 1.8 months in the warfarin group. Also, there are few drug interactions with DOACs, and they do not require any monitoring compared to warfarin.^19^

A study by Naymagon et al. assessed the efficacy of different anticoagulants for non‐cirrhotic PVT and found DOAC to have 30% more PVT resolution than warfarin. Furthermore, they found DOAC to have an almost three‐time better recanalization rate than warfarin and a less significant bleeding risk.^20^

The AASLD recommends local or systemic thrombolysis only in select cases of PVT suffering from intestinal ischemia despite anticoagulation therapy.^17^ The ACG recommends thrombolysis therapy in patients with progressive thrombosis despite anticoagulation treatment.^18^ In an observational study of 20 participants with acute mesenteric vein thrombosis with no improvement despite anticoagulation treated with thrombolysis, 19 out of 20 had recanalized the mesenteric vein.^21^

Thrombolysis is a relatively newer and sparsely used treatment modality compared to systemic anticoagulation for treating acute PVT, and different techniques of thrombolysis are evolving. A systematic review of 9 studies with 134 patients showed an 84% rate of recanalization and 86% improvement in symptomatology post‐thrombolysis, with only a 7% rate of major complications.^22^ Another study assessed the efficacy of catheter‐directed thrombolysis and found an 80% recanalization rate for non‐cirrhotic PVT with 2‐year patency to be 84%.^23^

Our patient presented with acute PV, SMV, and SV thrombosis, most likely secondary to the use of OCP. She remained symptomatic despite being initiated on LMWH. According to the ACG, patients with progressive thrombosis and an increased risk of intestinal ischemia could be considered for endovascular management. Combined portal vein and mesenteric vein thrombosis displayed on CT scans are independent predictors of transmural bowel necrosis.^24^ Our patient remained symptomatic despite therapeutic anticoagulation, and she was at increased risk of intestinal ischemia and infarction due to the combined portal‐mesenteric and splenic thrombosis. This led us to manage her successfully with thrombolysis and thrombectomy, with marked radiological and symptom improvement.

Portal vein thrombosis is a rare but severe condition most commonly seen in the cirrhotic population and less commonly in other illnesses with a prothrombotic state. Anticoagulation remains the mainstay of treatment in cirrhotic and non‐cirrhotic patients with PVT. Interventional therapies like thrombolysis and thrombectomy are relatively new methods of management with limited availability. It is reserved for patients with extensive thrombosis who remain symptomatic despite anticoagulation or those at risk of developing complications like mesenteric ischemia and infarction. Further prospective, case‐controlled studies are required to establish a standardized protocol, indication, route, and dosing of thrombolytic therapy and mechanical thrombectomy in PVT.

## AUTHOR CONTRIBUTIONS

**Mohammadshah Isam Gul:** Conceptualization; data curation; writing – original draft; writing – review and editing. **Waseem Umer:** Conceptualization; writing – original draft; writing – review and editing. **Muhammad Zahid:** Conceptualization; data curation. **Ahmed Daniyal Nawaz:** Conceptualization; data curation; writing – original draft; writing – review and editing. **Mohammad J. H. Elhissi:** Investigation; visualization.

## CONFLICT OF INTEREST STATEMENT

In compliance with the ICMJE uniform disclosure form, all authors declare no actual or potential conflict of interest.

## ETHICS STATEMENT

Consent was obtained or waived by all participants in this study. ABHATH issued approval MRC‐04‐22‐770.

## CONSENT

Written informed consent was obtained from the patient to publish this report in accordance with the journal's patient consent policy.

## Data Availability

Not available.
